# Effect of Stretching on the Electroencephalography of People With Irritable Bowel Syndrome

**DOI:** 10.7759/cureus.92709

**Published:** 2025-09-19

**Authors:** Toru Yasukawa, Yusuke Yamazato, Kaho Tanobe, Minori Machida, Toyohiro Hamaguchi, Jun Tayama

**Affiliations:** 1 Department of Rehabilitation, Graduate School of Health Sciences, Saitama Prefectural University, Saitama, JPN; 2 Graduate School of Human Sciences, Waseda University, Saitama, JPN; 3 Faculty of Human Sciences, Waseda University, Saitama, JPN

**Keywords:** default mode network, electroencephalography (eeg), ibs (irritable bowel syndrome), somatosensory processing, stretching

## Abstract

Introduction

Irritable bowel syndrome (IBS) is a functional gastrointestinal disorder characterized by chronic abdominal pain and altered bowel habits without structural abnormalities. Irritable bowel syndrome affects approximately 9.3% of the Japanese population based on Rome III criteria, with higher prevalence observed in younger adults, and is associated with significant impairments in quality of life. Its multifactorial pathophysiology involves dysregulation of the brain-gut-microbiome axis, with altered brain activity, such as reduced alpha and increased beta power on electroencephalography (EEG) during eyes-closed resting states.

Although nonpharmacological interventions such as abdominal and trunk stretching have demonstrated potential in reducing psychological stress markers, their neurophysiological effects in individuals with IBS remain poorly characterized. This study aimed to investigate the neurophysiological and somatosensory effects of a brief abdominal stretching intervention in patients with IBS. We hypothesized that stretching would (1) increase frontal alpha power and (2) increase somatic sensory and pain thresholds.

Methods

Fourteen adult males (mean age, 23.5 ± 1.2 years) with symptomatic IBS based on the Japanese version of the IBS Severity Index completed an Irritable Bowel Syndrome Quality of Life measure, followed by baseline assessments of sensory and pain thresholds and a 10-minute resting-state EEG. Participants performed a 4-minute supine trunk rotation stretching protocol involving bilateral 1-minute holds repeated twice per side until mild discomfort was perceived.

Alpha power (8-12.8 Hz) from EEG recordings and sensory and pain thresholds were statistically analyzed using paired-sample Student's *t*-tests to compare pre- and post-intervention data. Statistical significance was set at *p < *0*.*05.

Results

Alpha power in the frontal regions significantly increased after the stretching intervention (*t *(13) = 3.28, *p < *0*.*01, *d *= 0.31). Sensory thresholds also increased significantly after the intervention (*t *(13) = 2.76, *p *= 0.02, *d *= 1.04), while pain thresholds showed no significant change (*t *(13) = 0.98, *p *= 0.35, *d *= 0.37).

Conclusions

The increase in alpha power supports the hypothesis that stretching induces neurophysiological relaxation by modulating central sensory processing. The elevation in sensory thresholds may reflect reduced somatosensory hypersensitivity; the lack of change in pain thresholds suggests that the intervention did not substantially affect nociceptive processing, possibly due to limited stimulation intensity or duration. Collectively, these findings suggest that stretching may serve as a viable self-management strategy for IBS by modulating both EEG activity and somatosensory indices. Further randomized controlled trials are warranted to validate these effects and investigate their potential association with autonomic nervous system dynamics.

## Introduction

Irritable bowel syndrome (IBS) is a functional gastrointestinal disorder characterized by chronic abdominal discomfort and altered bowel habits in the absence of identifiable structural or biochemical abnormalities [1]. Given the well-established interplay between psychological and physiological factors in IBS [2], this condition is often regarded as a model disorder in psychosomatic medicine. Key pathophysiological features include psychological dysfunction, altered brain activity, and hypersensitivity to visceral stimuli, as reflected by reduced sensory thresholds [3]. In Japan, the estimated prevalence of IBS is 9.3% based on the Rome III criteria and 2.2% based on the more stringent Rome IV criteria [4]. Compared with healthy individuals, patients with IBS exhibit significantly lower quality of life (QOL), particularly in physical health, social functioning, and emotional well-being [5].

Current clinical guidelines recommend combining pharmacological treatment with non-pharmacological interventions for IBS [6]. Recent studies highlight the efficacy of cognitive behavioral therapy (CBT), dietary interventions, and physical activity in alleviating IBS symptoms and improving QOL [7]. Furthermore, self-management strategies that empower patients to take an active role in managing their condition have gained attention as effective adjuncts to conventional care [8].

Functional brain abnormalities have been observed in patients with IBS, including alterations in the default mode network (DMN) [9], which is a large-scale neural network that includes the medial prefrontal cortex, posterior cingulate cortex, precuneus, and the temporoparietal junction. The DMN is involved in self-referential thinking, memory retrieval, future planning, and emotional regulation, and is typically active during resting states with minimal external stimuli [10]. In patients with IBS, reduced DMN connectivity has been linked to heightened interoceptive attention, impaired emotional regulation, and hyperactivation of pain-related neural circuits [11]. These functional abnormalities were also reflected in electroencephalographic (EEG) findings. Some studies have reported decreased frontal alpha power and increased occipital beta power during resting-state EEG in patients with IBS [12], which may reflect dysregulation of the default mode network and altered regulation of psychological arousal.

Self-management approaches in IBS aim not only to control symptoms but also to promote patient autonomy, as empowering individuals to actively manage their symptoms has been associated with improved QOL [13]. Common self-management strategies include CBT, exercise therapy, and dietary interventions. Moss-Morris et al. conducted a randomized controlled trial (RCT) with 64 patients with IBS, implementing a 7-week CBT program with a self-management guidebook. This intervention, which included education on gastrointestinal physiology and stress, symptom monitoring, cognitive restructuring, and behavioral assignments related to diet, exercise, and relaxation, led to significant improvements in symptom severity and disease burden compared with controls [14].

Johannesson et al. conducted an RCT with 102 patients with IBS, providing individualized exercise recommendations (20-30 minutes per session, 3-5 days per week) and personalized goal setting. The intervention group showed a significant increase in physical activity and a notable reduction in IBS symptom severity compared with the control group [15]. More recently, Ankersen et al. demonstrated that both a low-FODMAP (Fermentable Oligosaccharides, Disaccharides, Monosaccharides, and Polyols) diet and probiotic treatment, delivered via an online platform, significantly alleviated IBS symptoms in 34 participants [16].

Exercise therapy has gained increasing attention as a self-management component for IBS [17]. An observational study involving 101 university students with IBS showed a significant inverse association between step count and scores on the Gastrointestinal Symptom Rating Scale (GSRS), suggesting that increased walking activity may relieve IBS symptoms [18]. Furthermore, an intervention study with 18 patients with IBS demonstrated that a 4-minute abdominal muscle stretching exercise significantly reduced salivary chromogranin A, a stress biomarker, and showed trends toward improved GSRS and State-Trait Anxiety Inventory scores [19].

In previous studies, the stretching exercise has been defined as a static trunk rotation stretch lasting approximately four minutes, which gently elongated the abdominal and trunk muscles and was reported to promote relaxation and autonomic regulation. These findings suggest that stretching may enhance parasympathetic activity, reducing stress responses and gastrointestinal symptoms in patients with IBS. Additionally, a study involving 19 healthy young adults found that stretching influenced brain activity and autonomic regulation via afferent somatosensory pathways, as reflected in changes in EEG and autonomic markers [20]. Collectively, these findings suggest that somatosensory input from stretching may normalize abnormal brain activity in patients with IBS.

In the present study, we employed a stretching protocol previously shown to reduce psychological stress in individuals with IBS [19]. Although stretching has been shown to alleviate stress in patients with IBS, its effects on neural activity, particularly as measured by EEG, have not been fully elucidated. This study aimed to investigate whether a stretching intervention in people with IBS would (1) increase alpha power in the frontal regions of the brain (Hypothesis 1) and (2) somatosensory thresholds, including both sensory and pain thresholds (Hypothesis 2).

A portion of this research in abstract form was presented at the 2024 Annual Meeting of the Japanese Society of Behavioral Medicine (February 1-2, 2025), the 2025 Conference of the Institute of Applied Brain Sciences at Waseda University (February 10, 2025), and the 2026 Annual Meeting of the Japanese Society of Psychosomatic Medicine (June 21-22, 2026).

## Materials and methods

Study design

This study utilized a single-group, pre-post intervention design that targeted people with IBS. The trial was registered with the University Hospital Medical Information Network Clinical Trials Registry on May 15, 2024 (Registration No. UMIN000055122; https://center6.umin.ac.jp/cgi-open-bin/ctr_e/ctr_view.cgi?recptno=R000062971). Ethical approval was obtained from the Institutional Review Board for Research Involving Human Subjects at Waseda University (Approval No. 2024-142). This study was conducted between August 6 and December 12, 2024.

Participants

Participants were recruited from a university classroom using a volunteer sampling method after verbal and written explanations of the study. Inclusion criteria were male adults with IBS symptoms, defined as a score of ≥75 on the Japanese version of the IBS Severity Index (IBS-SI-J) [21]. The exclusion criteria were as follows: (1) prior diagnosis or treatment of IBS; (2) diagnosis of any psychiatric disorder; (3) history of psychiatric or psychosomatic treatment; (4) current lower back or lumbar pain; and (5) ongoing treatment by an orthopedic specialist. Fourteen symptomatic male participants with IBS, aged 21-26 years (mean ± standard deviation [SD]: 23.5±1.2 years), met the eligibility criteria and provided written informed consent.

Abdominal muscle stretching

The stretching intervention followed the protocol described by Hamaguchi et al. [19]. Participants lay in a supine position with knees flexed while rotating their pelvis and trunk by approximately 90° or until mild discomfort was felt. Each posture was held for 1 minute and repeated on the opposite side. This sequence was performed twice per side for 4 minutes. A yoga mat was used for comfort, and all procedures were performed in an electromagnetically shielded room.

Main outcome

The primary outcome was frontal alpha band power (8-12.8 Hz), measured during eyes-closed resting-state EEG before and after the stretching intervention. Decreased alpha power is a well-documented neurophysiological characteristic of IBS at rest [14] and serves as a valid indicator for assessing intervention efficacy.

Secondary outcomes

Secondary outcomes included the Irritable Bowel Syndrome Severity Index (IBS-SI-J), Irritable Bowel Syndrome Quality of Life measure (IBS-QOL-J), and somatosensory thresholds (sensory and pain thresholds). The IBS-SI-J assesses five domains: abdominal pain severity and frequency, bloating, satisfaction with bowel habits, and daily life interference [22]. It consists of five items, each rated on a 0-100 scale, with total scores ranging from 0 to 500 (higher scores indicating more severe symptoms). The IBS-QOL-J includes 35 items across eight subscales, scored on a 5-point Likert scale and converted to a 0-100 score (higher scores indicating better condition-specific QOL) [22]. The eight subscales are dysphoria, interference with activity, body image, health worry, food avoidance, social reaction, sexual concerns, and relationships. These outcomes were selected based on previous evidence that patients with IBS exhibit heightened sensitivity to both visceral and somatic stimuli [23].

Sample size

Sample size estimation was based on a previous EEG-based non-pharmacological IBS study [24], which reported a significant increase in relative alpha power at the O2 electrode (Glass’s *Δ* = 0.62). Based on this finding, we conservatively estimated the effect size as Cohen’s *d* = 0.62, assuming comparable within-group variability. A power analysis using G*Power 3.1 (Heinrich Heine University, Düsseldorf, Germany) for a two-tailed paired Student's *t*-test (*α* = 0.05 and power = 0.95) indicated a requirement of 36 participants. However, assuming a larger effect size (*d* = 0.80), which is commonly used in exploratory trials involving patients with IBS, a minimum of 23 participants would suffice. Given the pilot nature and practical constraints of the current study, a final sample of 14 participants was deemed adequate to detect moderate-to-large effect sizes with over 80% power.

EEG recording and analysis

EEG signals were recorded using 31 active sponge-type electrodes positioned according to the international 10-10 system (AF7, Fz, F3, F7, F9, FC5, FC1, C3, T7, CP5, CP1, Pz, P3, P7, TP9, O1, Oz, O2, TP10, P8, P4, CP2, CP6, T8, C4, FC2, FC6, F10, F8, F4, AF8). Data were collected using the ActiCHamp Plus system (Brain Products GmbH, Gilching, Germany) at a 500 Hz sampling rate for 10 min under eyes-closed resting conditions before and after the intervention.

For pre-intervention analysis, a stable 1-minute segment (minutes 5-6) was selected. Post-intervention data were extracted at least 1 minute after stretching to avoid acute motion artifacts. EEG data were preprocessed using MATLAB R2023b (MathWorks, Inc., Natick, USA) and EEGLAB v2022.1 (Swartz Center for Computational Neuroscience, University of California San Diego, La Jolla, USA). A bandpass filter (1-40 Hz) was applied, and frequency transformation was performed using Fast Fourier Transform. Alpha power was quantified within the 8-12.8 Hz frequency range [14].

Somatosensory measurement

Somatosensory thresholds were assessed using a portable peripheral nerve stimulator (PNS-7000; Nihon Kohden Corporation, Tokyo, Japan). Stimulating electrodes were placed on the medial forearm of the dominant hand. Electrical stimuli began at 0 mA and increased in 0.01 mA increments. Sensory threshold was defined as the lowest current perceived, and pain threshold as the lowest current perceived as painful. Each threshold was measured three times and averaged for analysis.

Procedure

After initial screening with the IBS-SI-J and collecting demographic data, eligible participants completed the IBS-QOL-J questionnaire. Baseline somatosensory thresholds were measured, followed by EEG setup and a 10-minute eyes-closed resting-state EEG. During EEG and stretching, the lights were off, and external noise was minimized in a shielded room to maintain consistent experimental conditions. Participants were instructed to refrain from caffeine intake on the test day to avoid EEG interference. Following the baseline EEG, participants performed the 4-minute stretching routine, immediately followed by another 10-minute resting-state EEG recording. Somatosensory thresholds were reassessed at the end of the session.

Statistical analysis

Data were expressed as mean ± SD. Paired t-tests were used to compare alpha power, sensory thresholds, and pain thresholds before and after stretching. To assess temporal dynamics, the post-intervention EEG data were divided into nine 1-minute epochs (1-2, 2-3, ..., 9-10 min), and alpha power in each epoch was compared with the pre-intervention segment using paired Student's *t*-tests. A two-tailed significance level of *p* < 0.05 was employed for all tests. All statistical analyses were conducted using MATLAB. No missing data were reported, and all participants completed the intervention protocol.

## Results

Demographic data

Table 1 summarizes the demographic characteristics of the 14 people with IBS. All participants met the inclusion criteria, and none were excluded. The mean IBS-SI-J score was 147.8 ± 52.9, indicating moderate symptom severity. The mean IBS-QOL-J score was 91.0 ± 10.8, indicating relatively preserved QOL.

**Table 1:** Age, Gender, and inventory scores SD, standard deviation; IBS-SI-J, Japanese version of the Irritable Bowel Syndrome Severity Index; IBS-QOL-J, Japanese version of the Irritable Bowel Syndrome Quality of Life.

*N* = 14		Mean (SD)	Min	Max
Age in years		23.5 (1.2)	22	26
Gender (% male)		100.0	-	-
IBS-SI	Total score	147.8 (52.9)	90	263
	Abdominal pain (severity)	24.6 (26.4)	0	70
	Abdominal pain (duration)	18.6 (20.7)	0	80
	Abdominal distension	13.3 (22.6)	0	70
	Bowel movement	66.3 (26.3)	5	95
	Quality of life	25.1 (20.6)	0	65
IBS-QOL	Total score	91.0 (10.8)	55.2	100
	Dysphoria	89.0 (15.6)	37.5	100
	Interference with activity	87.1 (16.9)	32.14	100
	Body image	98.2 (5.0)	81.25	100
	Health worry	90.5 (17.2)	41.67	100
	Food avoidance	89.3 (13.1)	66.67	100
	Social reaction	96.3 (6.2)	81.25	100
	Sexual concerns	100.0 (0.0)	100	100
	Relationships	90.5 (19.6)	25	100

Primary outcome measure

**Figure 1:**
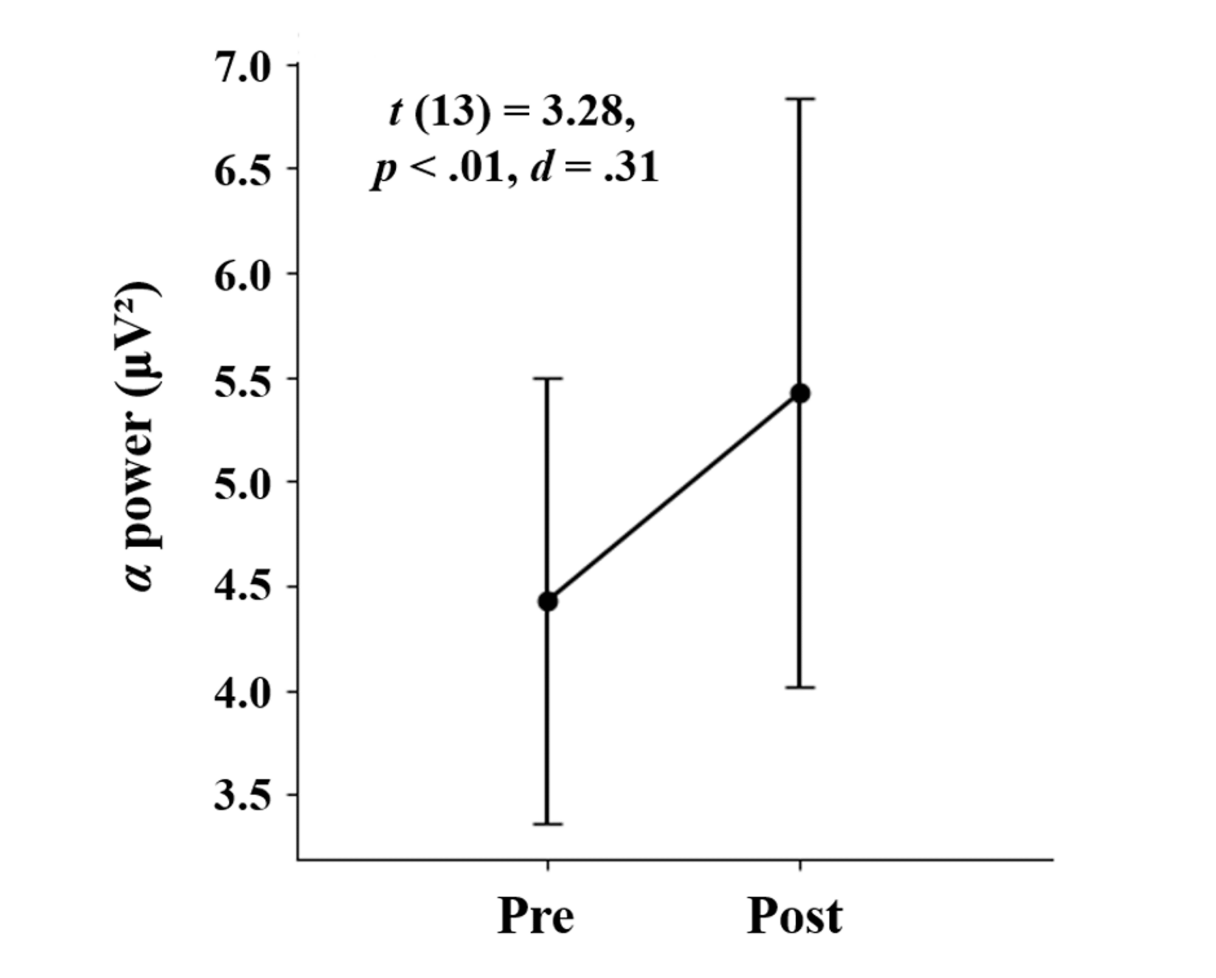
Alpha power in the frontal cortex at Pre and Post intervention Statistical analysis was conducted using Student’s *t*-test. The statistical significance was set at *p* < 0.05. The error bars represent standard errors.

Comparison of a stable 1-minute pre-intervention EEG segment with the first post-intervention segment revealed a significant increase in frontal alpha power (AT7, Fz, F3, F7, FC5, FC1, F8, F4, and AF8; *t* (13) = 3.28, *p* < 0.01, *d* = 0.31; Figure 1). Further analysis across nine consecutive 1-minute post-intervention segments revealed significantly elevated alpha power in all segments, except the sixth and ninth (Table 2). Although these two segments did not reach statistical significance, they showed marginal trends (*t* (13) = 1.86, *p* = 0.07, *d* = 0.18; *t* (13) = 1.95, *p* = 0.05, *d* = 0.18).

**Table 2:** Results of paired t-tests for frontal alpha power between Pre and Post intervention *p < 0.05, †*p* < 0.10

Analysis segment	*t*-value	*p*-value	Cohen's *d*
Segment 1 (1–2 min)	3.280	0.001*	0.310
Segment 2 (2–3 min)	3.329	0.001*	0.315
Segment 3 (3–4 min)	2.454	0.016*	0.232
Segment 4 (4–5 min)	2.761	0.007*	0.261
Segment 5 (5–6 min)	1.862	0.065†	0.176
Segment 6 (6–7 min)	3.903	0.000*	0.369
Segment 7 (7–8 min)	1.996	0.048*	0.189
Segment 8 (8–9 min)	3.013	0.003*	0.285
Segment 9 (9–10 min)	1.953	0.053†	0.185

Secondary outcome measure

**Figure 2:**
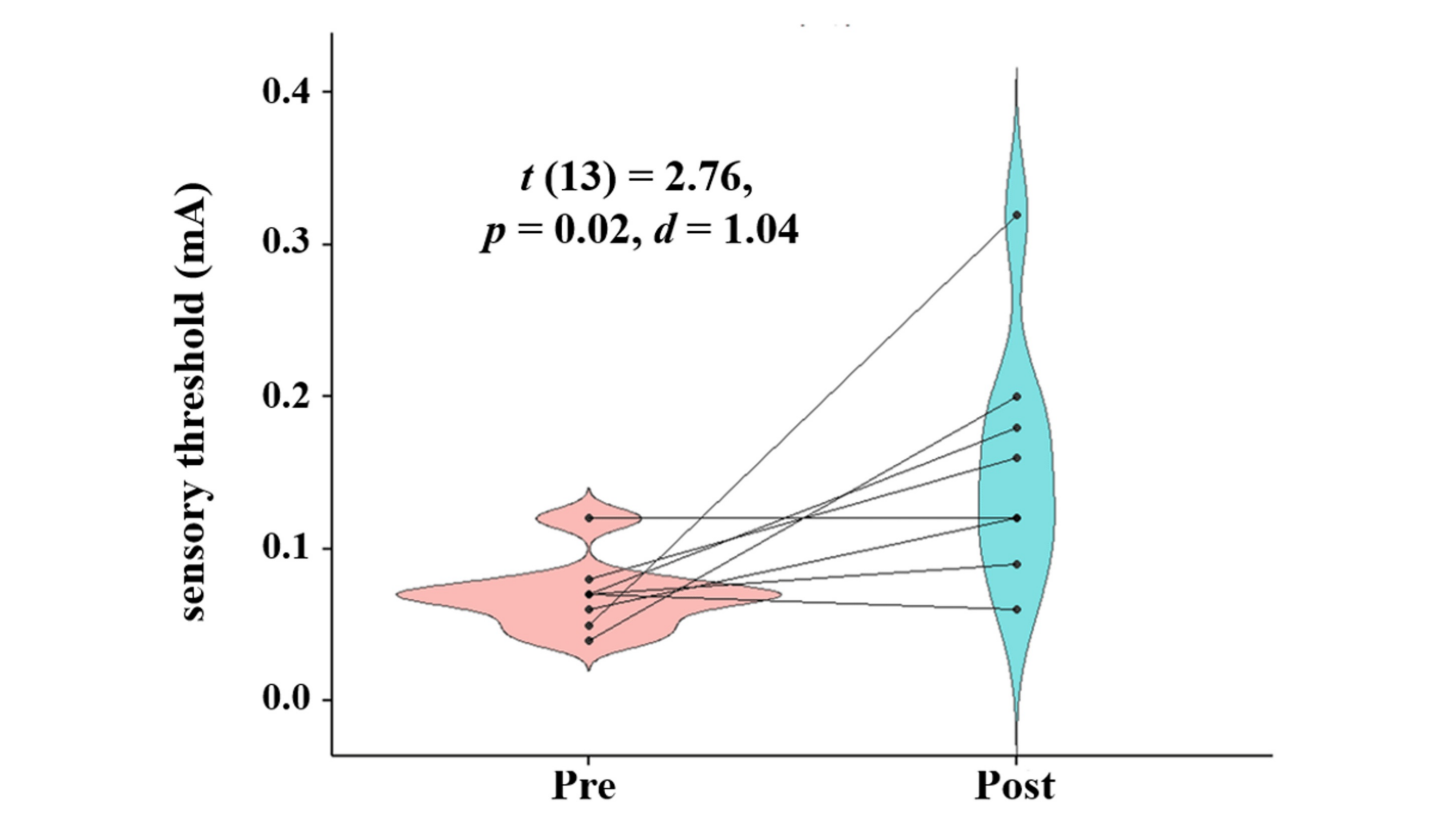
Sensory threshold at Pre and Post intervention Statistical analysis was conducted using Student’s *t*-test. The statistical significance was set at *p* < 0.05.

**Figure 3:**
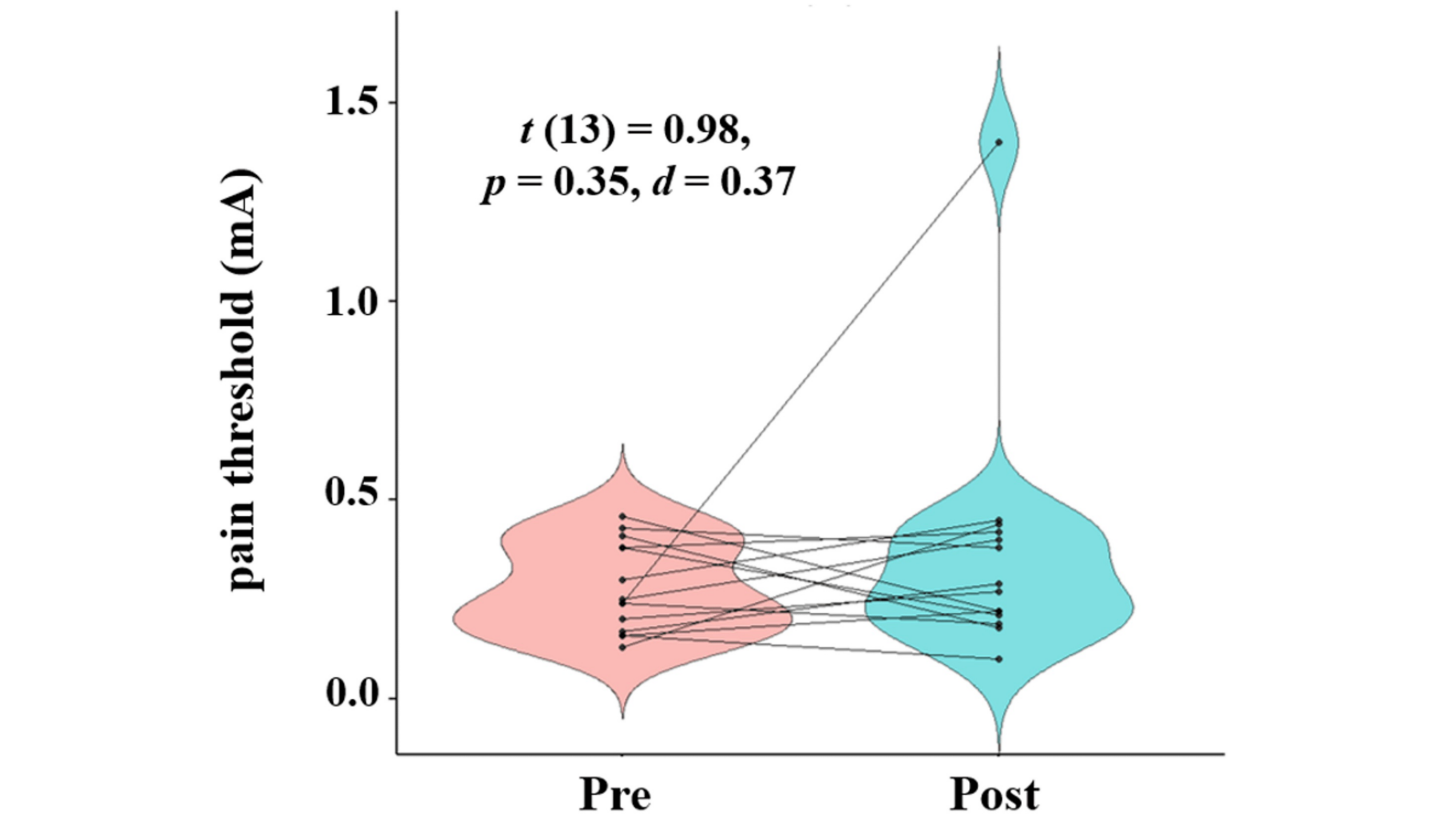
Pain threshold at Pre and Post intervention Statistical analysis was conducted using Student’s *t*-test. The statistical significance was set at *p* < 0.05.

Sensory thresholds significantly increased after the intervention compared to baseline (*t* (13) = 2.76, *p* = 0.02, *d* = 1.04), while no significant change was observed in pain thresholds (*t* (13) = 0.98, *p* = 0.35, *d* = 0.37). Table 3 summarizes the sensory and pain thresholds at baseline and post-intervention, while Figure 2 presents the pre- and post-intervention comparisons of sensory thresholds, and Figure 3 illustrates the corresponding changes in pain thresholds.

**Table 3:** Sensory and pain thresholds at baseline and post-intervention SD, standard deviation

Condition	Measure	Mean (SD)	Min	Max
Baseline (before stretching)	Sensory threshold	0.01 (0.06)	0.04	0.23
	Pain threshold	0.28 (0.11)	0.13	0.46
Post-intervention (after stretching)	Sensory threshold	0.16 (0.08)	0.06	0.32
	Pain threshold	0.37 (0.31)	0.10	1.40

## Discussion

This study investigated the effects of stretching on brain activity in people with IBS and evaluated the duration of these effects. The results support **Hypothesis 1**, demonstrating a significant post-intervention increase in frontal alpha power. **Hypothesis 2 **was partially supported; sensory thresholds significantly increased, while pain thresholds did not exhibit a statistically significant change.

Two mechanisms may explain the increase in frontal alpha power. First, sustained somatosensory input from stretching may have influenced central sensory processing. Hamaguchi et al. reported that strong visceral stimulation followed by weaker stimulation activated the midbrain, insular cortex, and cerebellum in healthy adults [25], suggesting that prior stimulation may induce central sensitization, whereas repeated input facilitates desensitization [26]. In this context, the somatosensory input from stretching may have temporarily attenuated central visceral reactivity, leading to an increase in alpha activity.

Second, stretching may have affected pain-processing systems that overlap with visceral processing, thereby indirectly modulating alpha power. Visceral and somatic sensations share a common neural network known as the neurological pain signature [27]. Somatosensory stimulation from stretching could downregulate hyperactive visceral processing and alleviate psychological tension, thereby increasing alpha activity. These findings suggest that stretching induces transient neuroplastic changes that influence brain activity.

The increase in sensory thresholds may reflect a reduction in somatosensory hypersensitivity and facilitation of central desensitization. A previous study involving 31 healthy adults reported that static stretching of the thigh increased sensory thresholds and reduced pain sensitivity [28]. Similarly, in the present study, afferent input from stretching may have modulated ascending sensory pathways, resulting in increased thresholds, consistent with prior evidence of somatosensory hypersensitivity in people with IBS.

The lack of significant change in pain thresholds may be attributed to two factors. First, sensory and pain thresholds are mediated by distinct neural mechanisms. Sensory thresholds are processed via low-threshold mechanoreceptors and the dorsal column-medial lemniscus pathway, while pain thresholds involve high-threshold nociceptors and the spinothalamic tract, projecting to regions such as the thalamus, insular cortex, and anterior cingulate cortex [29]. Therefore, stretching may have selectively influenced sensory but not nociceptive pathways. Second, the single 4-minute stretching session may have been insufficient to induce measurable changes in pain perception. More intensive or repeated interventions may be necessary to elicit changes in nociceptive thresholds.

Stretching is also known to activate the vagus nerve and attenuate sympathetic nervous system activity [22]. Given that people with IBS frequently display a sympathetic-dominant autonomic profile [30], stretching may have contributed to the restoration of autonomic balance and modulation of brain activity via the gut-brain axis [20]. However, this study did not include physiological measurements, such as heart rate or heart rate variability. Future investigations should incorporate autonomic nervous system assessments to elucidate the mechanisms by which stretching influences brain activity in patients with IBS.

This study had several limitations. First, the lack of a control group limited the ability to draw causal inferences. Future RCTs are needed to confirm the efficacy of the intervention. Second, the study focused exclusively on alpha activity, without examining other EEG frequency bands. Previous research has reported increased beta activity in patients with IBS [14], which is associated with heightened cognitive and stress-related processing. A more comprehensive EEG analysis is warranted. Third, somatosensory thresholds were assessed more than 10 minutes after the intervention, which may have introduced temporal variability. Although the observed increase in sensory thresholds was aligned with the increase in alpha power, immediate post-intervention measurements may better capture transient or acute effects. Finally, only a single, brief intervention session was tested. Future studies should examine the cumulative or sustained effects of repeated stretching interventions.

The strengths and implications of this study can be summarized in two main points. First, by restricting the sample to young male participants with symptomatic IBS, we were able to eliminate the potential confounding effects of age and sex, thereby enabling examination under more homogeneous conditions. Second, by assessing somatosensory function, the study demonstrated that stretching may desensitize the somatosensory hypersensitivity characteristic of IBS and improve sensory thresholds. Furthermore, the clinical significance of this study lies in the use of a simple and brief stretching procedure that can be easily incorporated into daily life, showing its potential to ameliorate core pathophysiological features of IBS. Collectively, these findings suggest that stretching may ultimately be established as a feasible self-management approach for patients with IBS.

## Conclusions

This study found that a brief trunk rotation stretching intervention was associated with a significant increase in frontal alpha power and somatosensory thresholds in individuals with IBS, though pain thresholds remained unchanged, suggesting differential modulation of sensory versus nociceptive pathways. These findings highlight the potential of stretching as a simple and feasible self-management strategy to ameliorate core pathophysiological features of IBS.

## References

[1] Mayer EA, Ryu HJ, Bhatt RR (2023). The neurobiology of irritable bowel syndrome. Mol Psychiatry.

[2] Hillestad EM, van der Meeren A, Nagaraja BH (2022). Gut bless you: the microbiota-gut-brain axis in irritable bowel syndrome. World J Gastroenterol.

[3] Mayer EA, Labus JS, Tillisch K, Cole SW, Baldi P (2015). Towards a systems view of IBS. Nat Rev Gastroenterol Hepatol.

[4] Sperber AD, Bangdiwala SI, Drossman DA (2021). Worldwide prevalence and burden of functional gastrointestinal disorders, results of Rome Foundation global study. Gastroenterology.

[5] Addante R, Naliboff B, Shih W, Presson AP, Tillisch K, Mayer EA, Chang L (2019). Predictors of health-related quality of life in irritable bowel syndrome patients compared with healthy individuals. J Clin Gastroenterol.

[6] Fukudo S, Okumura T, Inamori M (2021). Evidence-based clinical practice guidelines for irritable bowel syndrome 2020. J Gastroenterol.

[7] Tetali B, Suresh S (2024). Management of irritable bowel syndrome: a narrative review. Transl Gastroenterol Hepatol.

[8] Tayama J, Hamaguchi T, Koizumi K (2024). Efficacy of an eHealth self-management program in reducing irritable bowel syndrome symptom severity: a randomized controlled trial. Sci Rep.

[9] Zhao M, Hao Z, Li M (2023). Functional changes of default mode network and structural alterations of gray matter in patients with irritable bowel syndrome: a meta-analysis of whole-brain studies. Front Neurosci.

[10] Andrews-Hanna JR, Smallwood J, Spreng RN (2014). The default network and self-generated thought: component processes, dynamic control, and clinical relevance. Ann N Y Acad Sci.

[11] Jing C, Liu T, Li Q (2024). Study of dynamic brain function in irritable bowel syndrome via Hidden Markov Modeling. Front Neurosci.

[12] Fukudo S, Nomura T, Muranaka M, Taguchi F (1993). Brain-gut response to stress and cholinergic stimulation in irritable bowel syndrome. A preliminary study. J Clin Gastroenterol.

[13] Tayama J (2025). Self-management program for irritable bowel syndrome. Psychosom Med.

[14] Moss-Morris R, McAlpine L, Didsbury LP, Spence MJ (2010). A randomized controlled trial of a cognitive behavioural therapy-based self-management intervention for irritable bowel syndrome in primary care. Psychol Med.

[15] Johannesson E, Simrén M, Strid H, Bajor A, Sadik R (2011). Physical activity improves symptoms in irritable bowel syndrome: a randomized controlled trial. Am J Gastroenterol.

[16] Ankersen DV, Weimers P, Bennedsen M (2021). Long-term effects of a web-based low-FODMAP diet versus probiotic treatment for irritable bowel syndrome, including shotgun analyses of microbiota: randomized, double-crossover clinical trial. J Med Internet Res.

[17] Nunan D, Cai T, Gardener AD, Ordóñez-Mena JM, Roberts NW, Thomas ET, Mahtani KR (2022). Physical activity for treatment of irritable bowel syndrome. Cochrane Database Syst Rev.

[18] Hamaguchi T, Tayama J, Suzuki M (2020). The effects of locomotor activity on gastrointestinal symptoms of irritable bowel syndrome among younger people: an observational study. PLoS One.

[19] Hamaguchi T, Fukudo S, Kanazawa M, Tomiie T, Shimizu K, Oyama M, Sakurai K (2008). Changes in salivary physiological stress markers induced by muscle stretching in patients with irritable bowel syndrome. Biopsychosoc Med.

[20] Imagawa N, Mizuno Y, Nakata I (2023). The impact of stretching intensities on neural and autonomic responses: implications for relaxation. Sensors (Basel).

[21] Shinozaki M, Kanazawa M, Sagami Y (2006). Validation of the Japanese version of the Rome II modular questionnaire and irritable bowel syndrome severity index. J Gastroenterol.

[22] Kanazawa M, Drossman DA, Shinozaki M (2007). Translation and validation of a Japanese version of the irritable bowel syndrome-quality of life measure (IBS-QOL-J). Biopsychosoc Med.

[23] Wilder-Smith CH, Robert-Yap J (2007). Abnormal endogenous pain modulation and somatic and visceral hypersensitivity in female patients with irritable bowel syndrome. World J Gastroenterol.

[24] Tayama J, Saigo T, Ogawa S (2017). Effect of attention bias modification on brain function and anxiety in patients with irritable bowel syndrome: a preliminary electroencephalogram and psycho-behavioral study. Neurogastroenterol Motil.

[25] Hamaguchi T, Kano M, Kanazawa M, Itoh M, Yanai K, Fukudo S (2013). Effects of preceding stimulation on brain activation in response to colonic distention in humans. Psychosom Med.

[26] Naliboff BD, Berman S, Suyenobu B (2006). Longitudinal change in perceptual and brain activation response to visceral stimuli in irritable bowel syndrome patients. Gastroenterology.

[27] Van Oudenhove L, Kragel PA, Dupont P (2020). Common and distinct neural representations of aversive somatic and visceral stimulation in healthy individuals. Nat Commun.

[28] Støve MP, Hansen LØ, Elmbæk KK, Magnusson SP, Thomsen JL, Riis A (2025). The effect of stretching intensity on pain sensitivity: a randomized crossover study on healthy adults. Eur J Pain.

[29] Apkarian AV, Bushnell MC, Treede RD, Zubieta JK (2005). Human brain mechanisms of pain perception and regulation in health and disease. Eur J Pain.

[30] Chang L (2011). The role of stress on physiologic responses and clinical symptoms in irritable bowel syndrome. Gastroenterology.

